# Epidemiology and characteristics of repeat adverse drug reactions and associated clinical outcomes in older adults: a systematic review and meta-analysis

**DOI:** 10.1007/s41999-026-01501-2

**Published:** 2026-05-09

**Authors:** Behailu Terefe Tesfaye, Gregory M. Peterson, Woldesellassie M. Bezabhe, Olive M. Schmid, Mohammed S. Salahudeen

**Affiliations:** School of Pharmacy and Pharmacology, UTAS Health, University of Tasmania, P.O Box 845, Hobart, TAS 7006 Australia

**Keywords:** Adverse drug reaction, Repeat adverse drug reaction, Rehospitalisation, Older adults, Elderly, Drug safety

## Abstract

**Purpose:**

To systematically review and synthesise evidence on the incidence, characteristics, risk factors, and clinical outcomes of repeat adverse drug reactions (ADRs) in older adults.

**Methods:**

A comprehensive search of four major databases was conducted. Studies reporting data on both index (initial) and repeat ADRs in populations with a mean or median age ≥ 60 years were included. A repeat ADR was defined as the occurrence of the same or a different ADR following an index ADR in the same individual. A meta-analysis estimated the pooled incidence rate (IR) of at least one repeat ADR-related hospitalisation. The protocol was registered in PROSPERO (CRD420251086319).

**Results:**

Nine studies following participants with an index ADR to capture repeat ADRs and/or related hospitalisations were included. Seven examined repeat ADR-related hospitalisations, reporting incidences from 8.7 to 23.1% over 1–5 years. In six of the seven studies (n = 30,284) with pre-specified follow-up, the IR of at least one repeat ADR-related hospitalisation ranged from 4.0 to 13.0 per 100 person-year, with a pooled IR of 7.0 per 100 person-year (95% CI 5.0–10.0; I^2^ = 82.7%). Three studies found that the same drugs or drug classes were implicated in both index and repeat ADR-related hospitalisations in 41.7 to 60% of cases. In-hospital mortality among patients with repeat ADR-related hospitalisations ranged from 0 to 68.1% (3 studies).

**Conclusion:**

Repeat ADRs and related hospitalisations pose a non-trivial risk in older adults and often involve the same drugs. Future research should evaluate system-level strategies to reduce preventable repeat events.

**Supplementary Information:**

The online version contains supplementary material available at https://doi.org/10.1007/s41999-026-01501-2.

## Key summary points

**Aim:**

To synthesise evidence on the incidence, characteristics, risk factors, and clinical outcomes of repeat adverse drug reactions (ADRs) in older adults.

**Findings:**

Repeat ADR-related hospitalisations pose a clinically important burden among older adults, with a pooled IR of 7.0 per 100 person-year (6 studies). Repeat ADRs often involve the same drugs or drug classes as the initial event (3 studies). In-hospital mortality among patients with repeat ADR-related hospitalisation varied widely (3 studies).

**Message:**

Evaluation and implementation of enhanced ADR documentation and communication, comprehensive medication review, and targeted deprescribing may help reduce repeat ADRs and associated adverse outcomes.

**Supplementary Information:**

The online version contains supplementary material available at https://doi.org/10.1007/s41999-026-01501-2.

## Introduction

Adverse drug reactions (ADRs) pose a significant burden on patients and healthcare systems, contributing to increased morbidity, hospitalisations, mortality, and healthcare expenditure [1–4]. The burden of ADRs is pronounced among older adults [2, 5, 6]. In individuals aged 65 years and above, between 3.3 to 23.1% of hospitalisations are ADR-related [5], with a mean fatality rate estimated at 0.44% [2] compared with 0.2% in the general population [2]. The heightened vulnerability to ADRs among older adults is largely due to age-related physiological changes, frailty, and altered pharmacokinetics and pharmacodynamics, compounded by the high prevalence of multimorbidity and polypharmacy [7]. Despite recent improvements, older adults have historically been under-represented in clinical trials [8]. As a result, many medications prescribed in this population have not been adequately evaluated for safety or efficacy in this age group, increasing the risk of ADRs in real-world settings [8, 9].

Individuals with a history of an ADR are at elevated risk of experiencing further ADRs, which exacerbates the risk of adverse outcomes, including rehospitalisation and mortality [10, 11]. For instance, Schmid et al. reported that adults with an index ADR-related hospital admission had a nearly five-fold increased risk of another ADR-related admission within 90 days, and this elevated risk persisted for at least five years [11]. Similarly, Lopez et al. found that 8.8% of nursing home residents hospitalised for a first-time ADR were readmitted with a subsequent ADR within two years [12]. These hospitalisations may represent missed opportunities for intervention, with research suggesting that repeat ADRs often result from re-exposure to high-risk medicines, lack of communication across care transitions, or incomplete documentation of previous ADRs [13]. Unlike isolated ADRs, repeat ADRs often highlight systemic issues in medication management that extend beyond individual prescribers, settings and services [13, 14].

Previous systematic reviews have explored various aspects of ADRs in older adults, including risk prediction models [15], hospital admissions due to ADRs [5], the prevalence and characteristics of in-hospital ADRs [16, 17], and strategies to mitigate ADR risks [18]. However, while single ADR events have been well documented, repeat ADRs occurring after an index episode remain relatively underexplored. To our knowledge, no systematic review has specifically addressed the incidence of repeat ADRs and associated clinical outcomes, including mortality and hospitalisation. Additionally, there is a gap in reviews examining the availability, types, and effectiveness of interventions designed to prevent repeat ADRs. The present review therefore synthesised existing evidence on the incidence, characteristics (frequently implicated medications and ADR manifestations), risk factors, and clinical outcomes of repeat ADRs in older adults. Additionally, this review explored any available evidence on strategies or interventions intended to prevent repeat ADR episodes.

## Materials and methods

This systematic review and meta-analysis was conducted in accordance with the Cochrane methodological framework [19], and was reported following the Preferred Reporting Items for Systematic Reviews and Meta-Analyses (PRISMA) 2020 guideline [20]. The study protocol was registered in PROSPERO (CRD420251086319).

### Eligibility criteria

#### Inclusion criteria

We aimed to include randomised controlled trials and non-randomised studies, including quasi-experimental and observational studies, that explicitly reported information on both index (initial) and repeat ADR occurrence in adults with a mean or median age of 60 years and older, irrespective of the study setting (hospital, aged care/nursing home, outpatient, or community), ADR definition used, and tool employed to identify and assess an ADR. Within this review, an ADR was defined as any event explicitly described by the original study authors as an ADR, irrespective of the specific definition used in that study. A repeat ADR was defined as the occurrence of the same or different type of ADR in patients with an index ADR episode. Repeat ADRs could involve the same or different drugs or drug classes to those implicated in the index ADR. A repeat ADR-related hospitalisation was defined as a subsequent ADR-related admission occurring after an index ADR-related hospitalisation within a specified follow-up period. Only peer-reviewed studies involving human participants and published in English were included, with no restrictions on publication year.

#### Exclusion criteria

The following records were excluded: case reports, case series, conference abstracts or proceedings, theses, unpublished studies, and studies limited to a specific medical condition, drug or drug class.

### Information source and search strategy

Four major databases (Ovid Embase, Ovid Medline, International Pharmaceutical Abstracts (IPA), and PsycINFO) were systematically searched from their inception until July 9, 2025. The search was conducted in consultation with a subject librarian. The search strategy included the key terms ‘adverse drug reaction’, ‘repeat’, ‘readmission’, and ‘older adults’, along with their synonyms and subject headings. Boolean operators (AND, OR), truncation, wildcards and filters were applied as appropriate. Auto-alerts were set up in Ovid to capture newly-published relevant studies. Supplementary searches were conducted using Google Scholar, hand-searching the reference lists of included studies, and citation tracking (snowballing) via Web of Science. The full systematic search strategies for all databases are provided in the supplementary information (Table S1–S4).

### Study selection process

Search results were imported into Covidence [21] for screening. Title and abstract screening were independently performed by two reviewers (BT and GP/WB/OS/MS). Discrepancies were resolved through discussion and consultation with a third reviewer. The same procedure was applied to full-text screening.

### Data extraction process

Data were extracted by one reviewer (BT) and independently verified by a second reviewer (GP/WB/OS/MS). Extracted data included: citation details, country, participant demographics (sex, age), study design, data source, setting and population, follow-up duration, definitions and assessment methods for ADRs, and outcomes related to repeat ADRs.

### Outcomes and measurement

The outcomes were incidence of repeat ADRs, characteristics of repeat ADRs (implicated drug classes and resulting ADR manifestations), risk factors associated with repeat ADRs, and clinical outcomes following repeat ADRs (including mortality). Evaluated strategies or system-level interventions aimed at reducing repeat ADRs were considered exploratory secondary outcomes.

Repeat ADR-related hospitalisation was quantified using incidence proportion and incidence rate (IR). The incidence proportion was calculated by dividing the number of patients with at least one repeat ADR-related hospitalisation (new cases) by the number of patients with an index ADR-related hospital admission (population at risk). The IR was calculated by dividing the number of patients with at least one repeat ADR-related hospitalisation by the product of the number of patients with an index ADR-related hospital admission and the follow-up duration in years. Similar procedures were followed to calculate the incidence and IR of patients with at least one repeat ADR. In calculating the incidence proportion or IR of repeat ADR-related hospitalisation, loss to follow-up and mortality during follow-up timeframe were not accounted for; therefore, the estimates assume complete follow-up and do not account for the competing risk of death. 

### Data synthesis and analysis

A combination of qualitative synthesis and meta-analysis was used to summarise the outcomes. Drugs implicated in repeat ADRs were mapped to the Anatomical Therapeutic Chemical (ATC) Classification System level 2 [22]. Meta-analysis was conducted using STATA version 19 (StataCorp LLC). Prior to analysis, the IR of at least one repeat ADR-related hospitalisation was calculated for studies that reported both the follow-up duration and number of patients with a repeat ADR-related hospitalisation. A random-effects meta-analysis was performed using the Restricted Maximum Likelihood (REML) method to estimate between-study variance. Wald-type confidence intervals were used to determine the confidence interval (CI) for the overall summary effect. Heterogeneity across studies was assessed using Cochran’s Q and Higgins’ I^2^ statistics [23]. I^2^ values of 0%, 25%, 50%, and 75% were interpreted as indicating no, low, moderate, and high heterogeneity, respectively. Subgroup analysis was conducted based on the method used to identify repeat ADRs leading to hospitalisations. The methods used were extracting International Classification of Diseases (ICD) codes [24, 25] from administrative data, and clinical judgement combined with the Naranjo algorithm [26]. A two-sided p-value < 0.05 was considered statistically significant. We could not conduct meta-regression analysis and publication bias testing due to the limited number of studies included in the analysis (< 10) [23].

### Methodological quality assessment

The quality assessment of the studies was carried out by two independent reviewers (BT and GP/WB/OS/MS). Any disagreements between the reviewers were resolved through discussion and consensus, or by consulting a third author. The Joanna Briggs Institute (JBI) Critical Appraisal Checklist for Studies Reporting Prevalence Data or Incidence Data was used to evaluate the methodological quality of the included articles [27]. The checklist consists of nine elements that evaluate various aspects of each study, including the appropriateness of the sample frame, the method of sampling study participants, the sample size, and the data analysis techniques used. Additionally, the checklist evaluates the appropriateness of the methods for identifying and measuring conditions, adequacy of the response rate, and details on the description of the subjects and settings involved in the study. The JBI checklist provides four response options for each criterion: Yes, No, Unclear, and Not Applicable. The following cut-offs were used to assess the overall quality of individual studies: high quality, if 70% or more of the answers were ‘Yes’; moderate quality, if 50 to 69% of the answers were ‘Yes’; and low quality, if less than 50% of the answers were ‘Yes’ [28]. None of the eligible articles were excluded from the review based on the assigned quality scores.

### Certainty of evidence

The certainty of evidence for the pooled IR of at least one repeat ADR-related hospitalisation was evaluated using the Grading of Recommendations Assessment, Development and Evaluation (GRADE) system [29]. This framework assesses five key domains: risk of bias (ROB), inconsistency, indirectness, imprecision, and publication bias. Because of the small number of studies included in the review (< 10), we could not conduct a publication bias assessment; hence, the GRADE judgement was made based on the four other domains. The level of evidence for each outcome is classified into one of four categories: very low, low, moderate, or high [29]. A summary table was generated using the GRADEpro GDT software to present these findings [30]. The ROB associated with at least one repeat ADR-related hospitalisation was assessed using the risk of bias in non-randomised studies of exposure (ROBINS-E) tool [31]. This tool examines bias across seven domains: confounding factors, participant selection, exposure measurement, deviations from intended exposure, missing data, outcome measurement, and selective reporting. Each domain is rated as low ROB, some concerns, high ROB, or very high ROB. The overall ROB judgement is that for the domain with the greatest ROB. For instance, if the greatest ROB across domains is of high ROB, then the overall ROB at an individual study level is judged as at ‘high ROB’. The overall threat to conclusion is derived in a similar way to the overall ROB judgement at an individual study level. For example, if the greatest overall ROB at an individual study level is ‘high ROB’, then the threat to conclusion was considered high ROB [31]. High ROB was considered as ‘serious ROB’ while entering the overall ROB judgement into GRADEpro GDT.

## Results

### Study selection

**Fig. 1:**
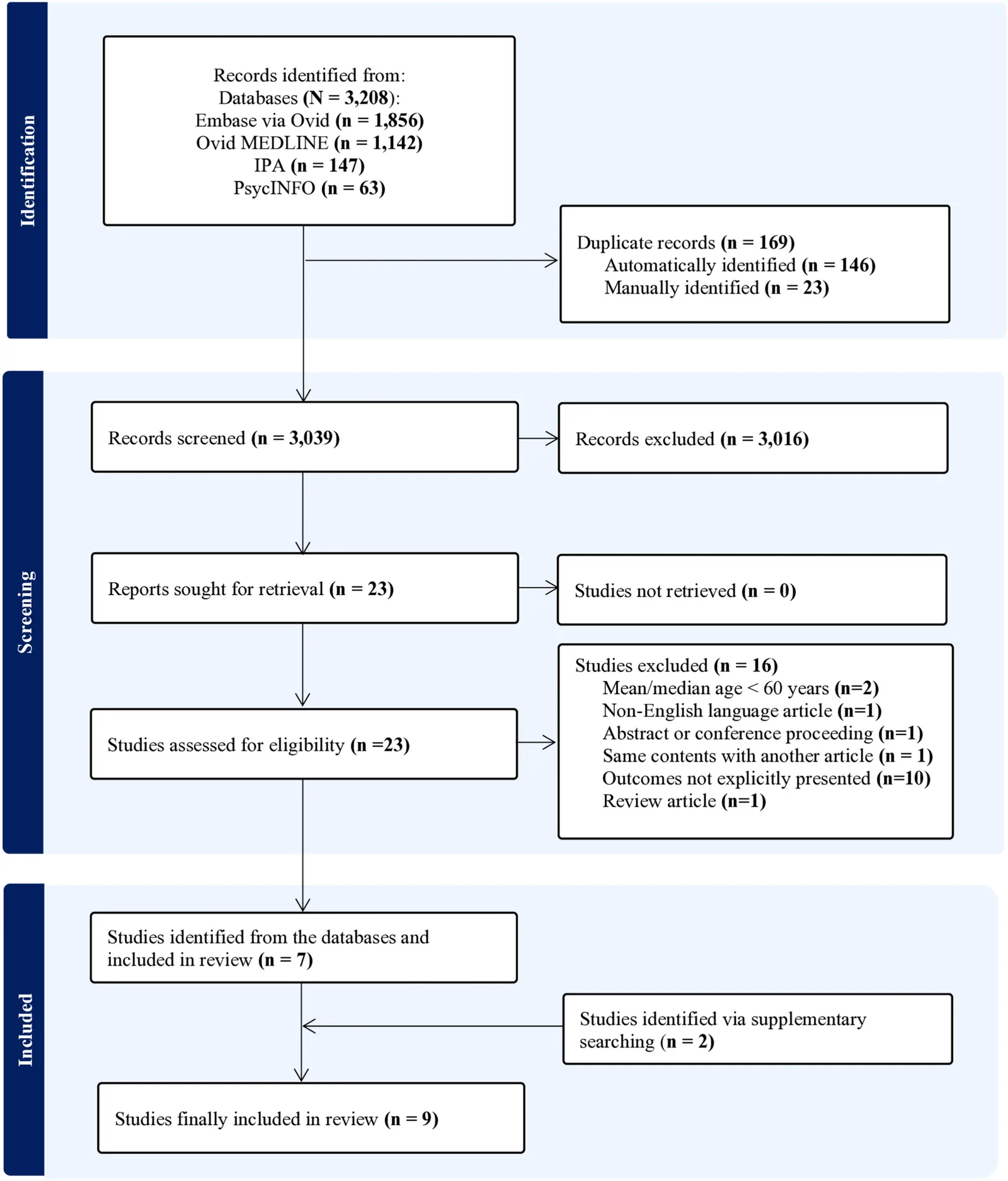
PRISMA flow diagram illustrating the study selection process for inclusion in this review

A systematic search of the four databases yielded a total of 3208 records. Following the removal of duplicates and screening of titles and abstracts, 23 studies were selected for full-text review. Additionally, two studies were identified through a supplementary search. Ultimately, nine studies met the eligibility criteria and were included in this review (Fig. 1).

### Patient and included study characteristics

Of the nine studies included in this review, 8 were retrospective cohort studies [10, 12, 13, 32–36], while the remaining one was prospective cohort [37]. No randomised controlled trials or quasi-experimental studies were identified. The studies were conducted in Australia [10, 35, 36], France [12, 33], the United States of America [37], the United Kingdom [32], Canada [13], and South Korea [34].

Eight studies [10, 12, 32–37] explicitly reported the number of participants with an index ADR and/or associated hospitalisation (total participants = 311,753), ranging from 52 [37] to 244,173 [34]. Follow-up duration was reported in seven of these studies [10, 32–37], ranging from 1 to 5 years. Administrative data were the most frequently used data source (5 studies) [10, 12, 33, 34, 36]. These five studies used the ICD codes recorded in the administrative data to identify ADRs [10, 12, 33, 34, 36], while clinical judgement by pharmacists [32, 35, 37] or a pharmacist and a physician [13], along with Naranjo causality assessment [26], was employed in the remaining four studies [13, 32, 35, 37]. Sex distribution data were extracted from five studies [10, 12, 33, 36, 37], all of which reported a higher percentage of females, ranging from 51.5% [33] to 90.4% [37]. Details of patient and study characteristics are shown in Table 1.

**Table 1:** Characteristics of patients and studies included in this systematic review

Study, year, (country)	Study design and data source	Setting	Female (%)	Age (years)	No. of participants	Follow-up duration (years)	ADR definition, identification, and assessment methods		
							ADR definition	ADR identification	Causality/severity/preventability/type
Lopez et al., 2025 (France) [12]	Retrospective cohort Administrative data	Toulouse University Hospital	68.7	Median 89.0 [IQR: 63.0,105.0]	409	2	WHO definition [38]	ICD-10 codes plus clinical judgement (Pharmacologists)	Preventability: Jonville-Bera [39] (11.1% preventable repeat ADR cases)
Hohl et al., 2019 (Canada) [13]	Retrospective cohort Previous research data	2 tertiary care centres and 1 urban community hospital	NR	Mean 66.0 (SD: 20.2)	NR	NR	WHO definition [38]	Clinical judgement (Pharmacist and Physician)	Causality: Modified Naranjo algorithm [26] Severity: Based on previous literatures [40–42] Preventability: Mixed approach (Results for repeat ADR causality, preventability, and severity: NR)
Lee et al., 2018 (South Korea) [34]	Retrospective cohort Administrative data	Tertiary and secondary hospitals, Primary care providers	NR	≥ 65	244,173	5	Customised based on ICD codes	ICD-10 codes (A1,12, B1, B2, and C)	Not assessed
Parameswaran Nair et al., 2017 (Australia) [35]	Retrospective cohort EHR	Royal Hobart Hospital	NR	≥ 65	112	1	WHO definition [38]	Clinical judgement (senior clinical pharmacist and consensus)	Causality: Naranjo algorithm [26] (All probable repeat ADRs) Severity: Hartwig severity scale [43] (All moderate repeat ADRs) Preventability: Modified Schumock and Thornton criteria [44] (73.3% preventable repeat ADRs) Type: Rawlins & Thompson [45] (All type A repeat ADRs)
Hauviller et al., 2016 (France) [33]	Retrospective cohort Administrative data	Toulouse University Hospital	51.5	Mean 77.6 (SD: 7.4)	1000	1	Edwards and Aronson definition [46]	ICD-10 codes	Preventability: Olivier’s scale [47] (22.2% preventable repeat ADR hospitalisations (index admission: non-chemotherapy ADR))
Davies et al., 2010 (United Kingdom) [32]	Retrospective cohort EHR and Previous research data	Royal Liverpool University Hospital	NR	Median 74 [IQR: 61–82]	163	1	Edwards and Aronson definition [46]	Clinical judgement (research pharmacist)	Causality: Naranjo algorithm [26] Severity: Hartwig severity scale [43] Preventability: Hallas Criteria [42] Types: Rawlins & Thompson [45] (Repeat ADR causality, severity, preventability, and type results: NR)
Zhang et al., 2009 (Australia) [36]	Retrospective cohort Administrative data	All hospitals in Western Australia (WA)	56.3	Median 72.4	28,548	3	Customised based on ICD codes	ICD-9 and ICD-9-CM (E930–E949) or ICD-10-AM (Y40–Y59) codes	Not assessed
Zhang et al., 2007 (Australia) [10]	Retrospective cohort Administrative data	Public and private hospitals in WA	56.1	Median 80	37 296	Mean 4.2 (SD: 4.3)	Customised based on ICD codes	ICD-9, ICD-9-CM, ICD-10-AM (Y40–Y59) codes	Not assessed
Cooper, 1999 (USA) [37]	Prospective Cohort Direct Patient Assessment	2 rural skilled-nursing facilities	90.4	Mean 81.4 (SD: 7.7)	52	4	Customised definition	Clinical judgement (consultant pharmacist)	Causality: Naranjo algorithm [26] (All probable repeat ADRs)

*ADR* Adverse drug reaction, *EHR* Electronic health record, *ICD* International classification of diseases *NHR* Nursing home residents, *WHO* World health organization

### Quality assessment

Methodological quality assessment of the included studies indicated that five studies were of high quality [10, 12, 33, 35, 36], one study was of moderate quality [32], and three studies were of low quality [13, 34, 37] (Table S5).

### Risk of bias assessment

ROB assessment for the outcome of at least one repeat ADR-related hospitalisation revealed that two studies had some concerns [33, 35] and four were rated as having high ROB [12, 32, 36, 37]. The overall ROB was rated as ‘serious’ (Table S6).

### Incidence, characteristics, and clinical outcomes of repeat ADRs

#### Incidence of repeat ADRs and clinical outcomes

**Fig. 2:**
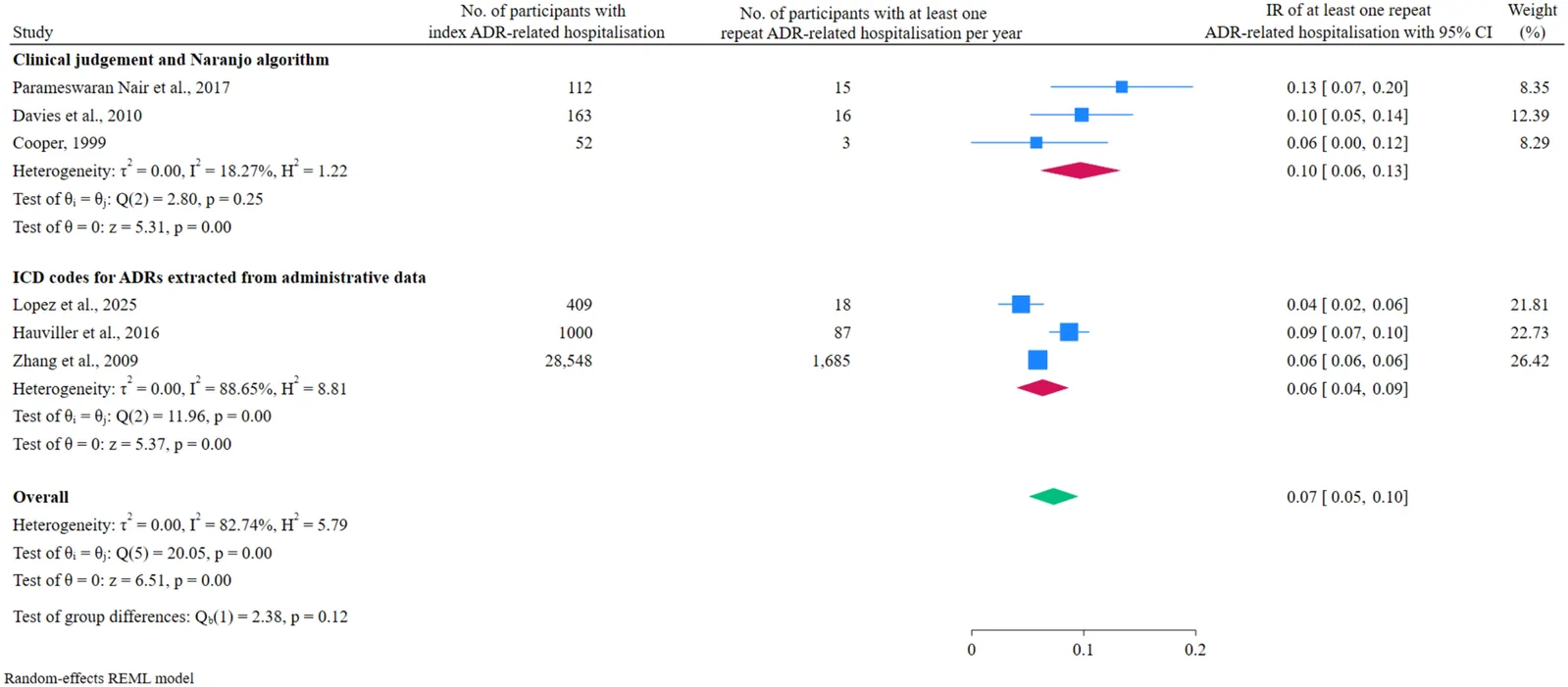
This forest plot visualises the IRs of at least one repeat ADR-related hospitalisation across individual studies and the pooled estimate, with subgroup analysis based on ADR identification methods. *ADR* Adverse drug reaction, *IR* Incidence rate

Of the nine studies included in this review, seven [10, 12, 32, 33, 35–37] investigated repeat ADR-related hospitalisations, with an incidence ranging from 8.7 to 23.1%. In the remaining two studies, the number of repeat ADRs or patients with repeat ADRs were reported, but it was not specified whether these events resulted in hospital admission [13, 34]. Among the seven studies evaluating repeat ADR-related hospitalisations, six [12, 32, 33, 35–37] stated a specific follow-up duration, as required for calculating the IR. In the six studies, the IR of at least one repeat ADR-related hospitalisation ranged from 4.0 (95% CI 2.0, 6.0) to 13.0 (95% CI 7.0, 20.0) per 100 person-year, with a pooled IR of 7.0 per 100 person-year (95% CI 5.0–10.0, I^2^ = 82.7%, n = 30,284, Low certainty). Leave-one-out meta-analysis showed that heterogeneity remained considerable (> 75%) across all iterations. The lowest heterogeneity (I^2^ = 75.1%) was observed when the study by Lopez et al. [12] was excluded. In subgroup analysis, studies that identified ADRs using ICD codes extracted from administrative data yielded a pooled IR of 6.0 per 100 person-year (95% CI 4.0–9.0) with high heterogeneity (I^2^ = 88.7%). In contrast, studies employing clinical judgement in conjunction with the Naranjo causality assessment method [26] yielded a pooled IR of 10.0 per 100 person-year (95% CI 6.0–13.0) with low heterogeneity (I^2^ = 18.3%) (Fig. 2, Table 2, and Table S7).

**Table 2:** The certainty of the pooled IR of at least one repeat ADR-related hospitalisation

No. of studies	Certainty assessment (IR of at least one repeat ADR-related hospitalisation)						Effect		Certainty
	Study design	Risk of bias	Inconsistency	Indirectness	Imprecision	Other considerations	No. of individuals	IR (95% CI)	
6 studies [12, 32, 33, 35–37]	Non-randomised studies	Serious^a^	Very serious^b^	Not serious	Not serious	All plausible residual confounding would reduce the demonstrated effect	30,284	7 per 100-person-year (5 to 10)	⨁⨁◯◯ Low^a,b^

^a^Mostly due to confounding (a critical domain in ROBINS-E) and selection of the participant related bias

^b^ There is considerable heterogeneity across the included studies as reflected by an I^2^ of 82.7% in Fig. 2

*ADR* Adverse drug reaction, *IR* Incidence rate

Of the two studies that reported repeat ADRs without specifying whether hospitalisation occurred [13, 34], Lee et al. reported repeat ADRs in 12.5% of participants who experienced an index ADR (30,520 people out of 244,173), corresponding to an IR of 2.5 per 100 person-year [34]. Hohl et al. did not specify the number of participants with index and repeat ADRs (some patients could have experienced more than one ADR simultaneously) [13].

In-hospital mortality among patients with repeat ADR-related hospitalisations was documented in three studies, with rates ranging from 0 to 68.1% [10, 33, 35]. In the study by Zhang et al. [10], although a crude cumulative mortality of 68.1% was reported among patients with repeat ADR-related hospitalisation, only 0.04% of deaths were attributed to an ADR overall. Meanwhile, Hauviller et al. reported that eight of the eleven patients with repeat ADR-related hospitalisations suffered chemotherapy-induced agranulocytosis [33].

#### Repeat ADRs and implicated drug classes

The manifestation of the repeat ADR associated with hospitalisation was reported in five studies [10, 12, 33, 35, 37]. The most common manifestations of repeat ADRs included aplasia/agranulocytosis (60.2%) [33], acute kidney injury (44.4%) [35], and falls (27.8%) [12]. Four of these five studies [12, 33, 35, 37], along with an additional study by Davies et al. [32], examined whether patient readmissions were associated with the same ADR as their index event. In four studies [12, 32, 35, 37], between one-quarter (4/15 = 26.7%) [35] and one-half (18/36 = 50%) [12] of readmissions were associated with the same ADR.

Among the five studies characterising the manifestation of repeat ADR-related hospitalisations [10, 12, 33, 35, 37], three [10, 33, 35] explicitly reported the most frequently implicated drug classes. Antineoplastic/immunosuppressive agents (11–76.4%) [10, 33], diuretics (0.4–44.8%) [33, 35], and drugs acting on the Renin–Angiotensin–Aldosterone System (RAAS) (31.0%) [35] were the most commonly associated drug classes. Additionally, four studies [10, 33, 35, 37] reported whether repeat ADR-related hospitalisations were associated with the same drugs/drug classes as the index hospital admission. In three of these studies [10, 35, 37], the proportion of repeat ADR-related hospitalisations associated with the same drugs linked to the index ADR ranged from 41.7% [37] to 60% [35]. Details of repeat ADRs and implicated drug classes are presented in Table 3 and Table S8.

**Table 3:** Summary of repeat ADRs, associated clinical outcomes, common implicated drug classes, and key risk factors

Study, year (country)	Key outcomes	Commonly implicated repeat ADRs	Commonly implicated medication classes	Risk factors for at least one repeat ADR-related hospitalisation
Lopez et al., 2025 (France) [12]	Patients with repeat ADR-related hospitalisations: 36 (8.8%); 50% due to same ADR	Falls (27.8%); haemorrhagic events (16.7%); constipation (11.1%); confusion (8.3%) Same ADRs in index and repeat admission: Falls (22.2%); constipation (16.7%); haemorrhagic events (11.1%)	NR	NR
Hohl et al., 2019 (Canada) [13]	Number of repeat ADRs: 132^a^	Constipation (9.8%), dizziness (7.5%), diarrhoea (7.5%), rash (6.0%), hyponatraemia (5.3%)	Diuretics (10.3%); antithrombotics (6.0%); analgesics (3.4%); corticosteroids (3.4%); RAAS agents (3.0%); antidiabetic drugs (3.0%)	NR
Lee et al., 2018 (South Korea) [34]	Patients with repeat ADRs: 30,520 (12.5%) Same preventable ADRs (57.5%) Same allergic reactions (42.5%) DUR intervention: ↓ Annual IR of repeat preventable ADRs ↑ Annual IR of repeat allergic reactions ↓ % of patients with repeat preventable ADRs post-DUR (2014) than pre-DUR (2010) (− 1.6% [–2.4, –0.8])	NR	NR	NR
Parameswaran Nair et al., 2017 (Australia) [35]	Patients with repeat ADR-related hospitalisations: 15 (13.4%) Total repeat ADRs: 18 Same ADR as index (26.7%) Same drug/class (60%) In-hospital mortality: None	Renal (44.4%); endocrine/ metabolic (27.8%); cardiovascular (16.7%); gastrointestinal (11.1%) disorders	Diuretics (44.8%), RAAS inhibitors (31.0%), drugs used in metabolic disorders (10.3%)	Higher PADR-EC scores at discharge (p = 0.034)
Hauviller et al., 2016 (France) [33]	Patients with repeat ADR-related hospitalisations: 87 (8.7%) Total ADRs: 259 In-hospital mortality (12.6%) Avoidable readmissions (non-chemotherapy group at index) (22.2%) Same drug and ADR (non-chemotherapy group at index): 10 cases	Aplasia/agranulocytosis (60.2%); polyneuropathy (12.0%); bleeding (6.2%); increased INR (3.5%); elevated serum digoxin level (1.5%)	Antineoplastic/immunosuppressive agents (76.4%); antithrombotics (7.9%); corticosteroids (2.4%); psycholeptics/psychoanaleptics (2.4%)	Cancer (aOR = 7.69 [4.59–12.88])
Davies et al., 2010 (United Kingdom) [32]	Patients with repeat ADR-related hospitalisations: 16 (9.8%) Same ADR as index (37.5%)	NR	NR	NR
Zhang et al., 2009 (Australia) [36]	Patients with repeat ADR-related hospitalisations: 5056 (17.7%)	NR	NR	Male sex; private hospital; stay ≥ 14 days; admission 1995–1999; CCI ≥ 7; metropolitan residence; use of hormones, antineoplastics, immunosuppressives, bacterial vaccines; multiple comorbidities (cardiac, vascular, pulmonary, hepatic, renal, diabetic, malignant, neurologic)
Zhang et al., 2007 (Australia) [10]	Patients with repeat ADR-related hospitalisations: 6853 (18.4%) In-hospital mortality: 68.1% (repeat ADR admission) vs. 59.3% (non-ADR readmission), p < 0.001 ADR episodes linked to the same drug class: 4516 (44.2%)	Nausea/vomiting (8.0%); haemorrhagic disorders (5.5%); drug-induced osteoporosis (4.8%); poisoning by cardiovascular agents (3.9%); dermatitis (3.5%)	Antineoplastic/immunosuppressive agents (11.0%); corticosteroids (10.1%); antithrombotics (7.9%); analgesics (10.0%)	NR
Cooper, 1999 (USA) [37]	Patients with repeat ADR-related hospitalisations: 12 (23.1%) Patients with the same ADR in index and repeat hospitalisations: 5 (41.7%)	Same ADRs in index and repeat hospitalisations: NSAID gastropathy (3); NSAID-associated bleeding (2); oversedation due to CNS depressants (1)	NR	NR

^a^The number of participants with repeat ADRs and the follow-up duration were not reported

*ADR* Adverse drug reaction, *aOR* Adjusted odds ratio, *CCI* Charlson comorbidity index, *CNS* Central nervous system, *INR* International normalized ratio, *NR* Not reported, *NSAID* Nonsteroidal anti-inflammatory drug, *RAAS* Renin–angiotensin–aldosterone system

#### Risk factors for repeat ADRs

Factors associated with repeat ADR-related hospitalisation were identified in three studies [33, 35, 36]. Cancer was reported as a significant risk factor in two of these studies [33, 36]. In the study by Parameswaran Nair et al., patients who experienced repeat ADR-related hospital admissions had significantly higher PADR-EC (Prediction of Hospitalization due to Adverse Drug Reactions in Elderly Community-Dwelling Patients) scores at discharge from their index admission compared to those not readmitted for ADRs [35]. Additional patient characteristics associated with increased risk of repeat ADR-related hospitalisations included a Charlson Comorbidity Index score [48] ≥ 7, length of index hospital stay ≥ 14 days, and specific comorbidities at the time of index admission, such as diabetes with chronic complications and renal disease [35]. The details are shown in Table 3.

#### Strategies to reduce repeat ADRs

No interventional studies specifically evaluating strategies to reduce repeat ADRs were identified. However, one observational interrupted time-series study [34] assessed changes in repeat preventable ADRs before and after implementation of a national prospective drug utilisation review (DUR) programme. In this study, all ADRs with the exception of allergic reactions were deemed preventable. The DUR program integrated a systematic medication review with electronic health records (EHR) and insurance claims systems to enhance medication safety. Among individuals aged ≥ 65 years, DUR was associated with a gradual reduction in preventable repeat ADRs over five years (slope change: − 0.006; p ≤ 0.01). A comparison of data from the pre-DUR (2010) and post-DUR (2014) periods revealed a 1.6% relative reduction in the proportion of patients experiencing repeat preventable ADRs (Table 3).

## Discussion

To the best of our knowledge, this is the first systematic review and meta-analysis to synthesise evidence on repeat ADRs and their associated clinical outcomes in older adults. Across studies that reported sex distribution among participants with an index ADR, the majority were female [10, 12, 33, 36, 37]. As none specified sex-based inclusion criteria, this pattern likely reflects underlying demographic and biological factors. Women generally have a longer life expectancy [49], greater medication exposure and higher susceptibility to ADRs [50], and they are more likely to seek healthcare than men [50]. Their overrepresentation in residential aged care [49, 51], may also explain the high proportion of female participants seen in nursing home-based studies [12, 37].

Our pooled estimate indicates that at least one repeat ADR-related hospitalisations occurs at a rate of 7 per 100 person-year among older adults following an index ADR-related hospitalisation, based on studies with heterogeneous follow-up periods. However, the confidence in the estimate is low, influenced by the limited number of studies, variability in study quality, potential confounders, and substantial heterogeneity. The heterogeneity may have arisen from differences in ADR identification methods, the types of ADRs investigated, variations in patient populations, and other study-specific factors. To statistically explore the sources of heterogeneity, a subgroup analysis was conducted based on ADR identification methods. The results revealed low heterogeneity among studies using clinical adjudication with the Naranjo tool, and considerable heterogeneity among those relying on ICD codes extracted from administrative data. The variation in ICD coding practices across studies within the latter group may explain the observed difference in estimates, as shown in Table 1. For example, Zhang et al. [10] used ICD-9, ICD-9-CM, and ICD-10-AM (Y40–Y59) codes, whereas Lopez et al. [12] employed ICD-10 codes related to drug-induced ADRs. This explanation is supported by the evidence indicating that the sensitivity of ICD-based ADR detection varies depending on coding criteria used, ranging from 6.8 to 28.1% [25].

The higher pooled IR of at least one repeat ADR-related hospitalisation in studies utilising clinical adjudication (10.0 per 100 person-year) compared to those relying on ICD codes to identify ADRs (6.0 per 100 person-year) supports the evidence that clinical adjudication methods generally exhibit greater sensitivity in detecting ADRs than ICD-based approaches [52]. This suggests that reliance solely on administrative data may underestimate the true burden of repeat ADR-related hospitalisations, reinforcing the value of clinician review or adjudication in enhancing the accuracy of ADR surveillance. In this subgroup analysis, although a numerical difference was observed, the larger number of participants in the ICD-based studies compared with the clinical adjudication–based studies may have limited the power to detect a statistically significant difference between the approaches.

The persistence of susceptibility to ADRs following an index event emphasises the need for ongoing vigilance and proactive management, akin to approaches used for chronic conditions. One key contributor to repeat ADRs is unintentional re-exposure to drugs or drug classes previously identified as harmful for the patient [13], presenting an important opportunity for risk reduction. This is illustrated by a Canadian cohort study of 849 participants, in which 45.2% of patients diagnosed with adverse drug events (ADEs) were re-dispensed the culprit medication within one year of hospital discharge [53]. In line with these observations, three studies included in this review [10, 35, 37] found that the same drugs or drug classes were associated with index and repeat ADR-related hospitalisations in 41.7 to 60% of cases. While re-exposure may sometimes be clinically justified, particularly when therapeutic alternatives are limited or the anticipated benefits outweigh the risks, it underscores the need for strategies to reduce preventable repeat ADRs. Systematic documentation of newly identified ADRs and integration into the patient’s medical history [54] is a recommended strategy to reduce preventable ADRs. This approach alerts clinicians to prior ADRs when prescribing, dispensing, or administering medications, thereby reducing the risk of repeat events. Existing evidence also suggests that an interoperable EHR system across healthcare settings can further strengthen ADR risk prevention. Because patients often receive care from multiple providers, interoperability enables comprehensive access to medical histories, facilitating the identification of individuals at high risk of medication-related problems and adverse outcomes [55, 56]. However, given that repeat ADRs were associated with drugs different from those implicated in index ADRs in a comparable percentage of older adults, as indicated in the studies [10, 35, 37], interventions based solely on a patient’s prior ADR history may have potentially limited effect. In such cases, ADRs may be more closely related to multimorbidity, polypharmacy, and evolving therapeutic needs due to changes in clinical status.

Importantly, ADRs are multifactorial, arising from therapy-related inevitable risks [57, 58], underlying patient susceptibility [7], changes in clinical status, evolving medication regimens, and healthcare system factors, such as gaps in medication management, transitions of care, and inadequate risk stratification [14]. A previous study reported that interventions such as medication reconciliation during care transitions and medication optimisation reduced ADR-related hospital encounters [59]. However, not all ADRs are preventable. Certain medications, such as chemotherapy, may pose unavoidable risks due to their mechanism of action, even when appropriately managed [57, 58]. While targeting cancer cells, chemotherapy also destroys rapidly dividing normal cells, such as bone marrow, often leading to ADRs like agranulocytosis [57, 58]. In these contexts, repeat ADRs may occur despite appropriate management, and careful monitoring and multidisciplinary decision-making are essential to balance efficacy with tolerability. In this review, cancer was identified as a risk factor for repeat ADR-related hospitalisations in two studies [33, 36]. While this increased risk has been observed across age groups [60], older adults with cancer may face additional vulnerabilities due to reduced bone marrow reserve, impaired renal and hepatic function [61], multimorbidity, altered pharmacokinetics and pharmacodynamics, and limited safe therapeutic alternatives [7, 62].

The observed variability in in-hospital mortality rates (0–68.1%) among patients with repeat ADR-related hospitalisations across the three studies reporting this outcome [10, 33, 35] may be attributed to differences including population characteristics, study design, and ADR severity, among other factors. In the cohort examined by Parameswaran Nair et al. [35], no ADRs associated with cytotoxic drugs were reported, which may have contributed to a lower mortality risk, as these medications and patient groups are considered high-risk [63]. Additionally, serious ADRs are known to increase in-hospital mortality [64]; while severity was not assessed in two studies [10, 33], all repeat ADRs in the study by Parameswaran Nair et al. [35] were rated moderate. However, the finding that only 0.04% of overall mortality was attributed to ADRs in the study by Zhang et al. [10], suggests that the burden of mortality observed among patients with repeat ADR-related hospitalisations may not be directly caused by the ADRs themselves, but rather reflects underlying patient vulnerability and the broader clinical context, such as cancer complications.

This review is not without limitations. Heterogeneity among the included studies may have influenced the pooled IR estimates of repeat ADR-related hospitalisations. Eight [10, 12, 13, 32–36] studies included in this review were retrospective in design and most relied on administrative data [10, 12, 33, 34, 36] as their data source, which increases the likelihood that some patient information, particularly ADRs, was under-recorded or missed. Additional limitations include the potential omission of relevant ADR-related ICD-10 codes [12], possibility of hospitalisations to a setting not in-scope for the studies [12, 33], and potential variability in patient follow-up practice and ADR ascertainment. Furthermore, some of the studies included for estimating incidence and IR of repeat ADR-related hospitalisation lacked detailed information on patients who died and/or were lost to follow-up between the index and repeat ADR-related hospitalisations [12, 32, 37]; consequently, all the estimates assume complete follow-up and do not account for death as a competing risk. Collectively, these factors may have influenced estimates of repeat ADRs and related hospitalisations. A further limitation pertains to the lack of information about the specific drugs responsible for ADR-related hospital admissions in studies that used ICD codes, as stated by Zhang et al. [10]. Although this review targeted older adults, the use of a median or mean age of ≥ 60 years as an inclusion criterion may have led to the inclusion of studies that partially enrolled participants younger than 60 years, thereby limiting the generalisability of the findings to the intended population. Additionally, restricting inclusion to articles published in English may have introduced bias. Despite employing a comprehensive search strategy with controlled vocabulary, a broad set of ADR-related keywords, and supplementary searches, some relevant studies may still have been missed. Finally, because fewer than ten studies met the inclusion criteria, meta-regression analysis and formal assessment of publication bias could not be performed.

### Implications and future research

Frequent re-exposure to drugs implicated in index ADRs, as reported in some of the included studies, may reflect gaps in the continuity of medication information across primary care, hospitals, and aged care facilities, as well as the clinical necessity of using medications with a high benefit–risk profile. Broader ADR research suggests that strengthening comprehensive medication management at discharge and during follow-up, alongside interoperable ADR documentation and alert systems within EHRs, may help mitigate ADR-related adverse outcomes [54–56, 59]. Nevertheless, interventional evidence specifically addressing repeat ADRs remains limited. Only one observational study evaluated the impact of an existing DUR program and reported a reduction in repeat preventable ADRs. Future research should prioritise the development, implementation, and evaluation of targeted strategies to reduce repeat ADRs. Our review also indicates that risk factors for repeat ADRs and their associated clinical outcomes are not consistently identified across studies. Large-scale linked datasets and emerging analytical approaches, such as machine learning, may help develop robust predictive models for repeat ADRs and their outcomes. Finally, the broader consequences of repeat ADRs, including impacts on quality of life, functional and cognitive decline, caregiver burden, and healthcare costs, remain underexplored and warrant further investigation.

## Conclusions

The review showed that repeat ADRs and related hospitalisations represent a non-trivial burden among older adults. These events often involved the same drugs, drug classes, and/or ADRs as the index event, indicating a potential for preventability in many cases. Although not directly examined in this review, these patterns imply that strengthening processes for recording and communicating index ADRs across care settings, particularly during transitions such as hospital discharge, may help to reduce avoidable re-exposure and subsequent harm. Future research should prioritise the development and evaluation of system-level interventions, including interoperable medication records, robust ADR alert mechanisms, and predictive decision-support tools to identify high-risk individuals.

## Supplementary Information

Below is the link to the electronic supplementary material.

Supplementary file1 (DOCX 3594 KB)

## Data Availability

The datasets supporting the conclusions of this article are provided within the main text and its supplementary (S) information files.

## Code availability

No custom code was developed for this systematic review. All analyses were performed using standard functions available in STATA version 19 (StataCorp LLC).
