# Supplementary material for: Epidemiology and characteristics of repeat adverse drug reactions and associated clinical outcomes in older adults: a systematic review and meta-analysis
Source: Eur Geriatr Med. 2026 May 9;17(4):1841–56. doi: 10.1007/s41999-026-01501-2 (PMC13562334; doi:10.1007/s41999-026-01501-2)
Supplement: Supplementary file 1 [file 41999_2026_1501_MOESM1_ESM.docx]

**Supplementary information**

**Journal name**: [**European Geriatric Medicine**](https://link.springer.com/journal/41999)

**Article title**: Epidemiology and Characteristics of Repeat Adverse Drug Reactions and Associated Clinical Outcomes in Older Adults: A Systematic Review and Meta-Analysis

**Authors**: Behailu Terefe Tesfaye*, Gregory M. Peterson, Woldesellassie M. Bezabhe, Olive M. Schmid, Mohammed S. Salahudeen

School of Pharmacy and Pharmacology, UTAS Health, University of Tasmania, P.O Box 845, TAS 7006, Hobart, Australia

*Corresponding author: Behailu Terefe Tesfaye. Email: [behailuterefe.tesfaye@utas.edu.au](mailto:behailuterefe.tesfaye@utas.edu.au)

**Table S1** Systematic search strategies from Ovid MEDLINE(R)

| **#** | **Query** | **Results from 08 Jul 2025** |
| --- | --- | --- |
| 1 | "repeat*".ab,kf,ti. | 699,661 |
| 2 | recurrent.ab,kf,ti. | 375,483 |
| 3 | re?exposure.ab,kf,ti. | 1,226 |
| 4 | subsequent.ab,kf,ti. | 716,091 |
| 5 | post?index.ab,kf,ti. | 448 |
| 6 | Patient Readmission/ | 25,690 |
| 7 | "re?admission*".ab,kf,ti. | 50,304 |
| 8 | "re?hospital*".ab,kf,ti. | 9,702 |
| 9 | "re?visit*".ab,kf,ti. | 49,803 |
| 10 | "revolv*".ab,kf,ti. | 5,831 |
| 11 | 1 or 2 or 3 or 4 or 5 or 6 or 7 or 8 or 9 or 10 | 1,839,188 |
| 12 | "Drug-Related Side Effects and Adverse Reactions"/ | 40,545 |
| 13 | "adverse drug reaction*".ab,kf,ti. | 23,696 |
| 14 | "Drug related side effect*".ab,kf,ti. | 2,359 |
| 15 | "adverse drug effect*".ab,kf,ti. | 1,991 |
| 16 | "drug-related adverse reaction*".ab,kf,ti. | 225 |
| 17 | "adverse drug event*".ab,kf,ti. | 6,277 |
| 18 | 12 or 13 or 14 or 15 or 16 or 17 | 65,868 |
| 19 | exp Aged/ | 3,718,809 |
| 20 | aged.ab,kf,ti. | 848,425 |
| 21 | "old*".ab,kf,ti. | 2,007,953 |
| 22 | "geriatric*".ab,kf,ti. | 87,530 |
| 23 | senior*.ab,kf,ti. | 58,907 |
| 24 | "elder*".ab,kf,ti. | 340,901 |
| 25 | advanced age.ab,kf,ti. | 23,752 |
| 26 | 19 or 20 or 21 or 22 or 23 or 24 or 25 | 5,768,252 |
| 27 | 11 and 18 and 26 | 1,354 |
| 28 | limit 27 to (english language and humans) | 1,142 |

**Table S2** Systematic search strategies from Ovid Embase

| **#** | **Query** | **Results from 08 Jul 2025** |
| --- | --- | --- |
| 1 | repeat*.kf,ti. | 105,743 |
| 2 | recurrent.kf,ti. | 161,128 |
| 3 | re?exposure.kf,ti. | 125 |
| 4 | subsequent.kf,ti. | 37,662 |
| 5 | post?index.kf,ti. | 0 |
| 6 | hospital readmission/ | 123,197 |
| 7 | re?admission*.kf,ti. | 23,160 |
| 8 | re?hospital*.kf,ti. | 2,415 |
| 9 | re?visit*.kf,ti. | 34,082 |
| 10 | revolv*.kf,ti. | 578 |
| 11 | drug reexposure/ | 882 |
| 12 | 1 or 2 or 3 or 4 or 5 or 6 or 7 or 8 or 9 or 10 or 11 | 460,799 |
| 13 | adverse drug reaction/ | 376,278 |
| 14 | adverse drug reaction*.kf,ti. | 16,003 |
| 15 | Drug related side effect*.kf,ti. | 1,873 |
| 16 | adverse drug effect*.kf,ti. | 562 |
| 17 | drug-related adverse reaction*.kf,ti. | 46 |
| 18 | adverse drug event*.kf,ti. | 3,777 |
| 19 | 13 or 14 or 15 or 16 or 17 or 18 | 389,079 |
| 20 | exp aged/ | 4,266,457 |
| 21 | aged.kf,ti. | 116,860 |
| 22 | old*.kf,ti. | 374,820 |
| 23 | Elder*.kf,ti. | 233,964 |
| 24 | geriatric*.kf,ti. | 57,575 |
| 25 | senior*.kf,ti. | 12,533 |
| 26 | advanced age.kf,ti. | 2,609 |
| 27 | 20 or 21 or 22 or 23 or 24 or 25 or 26 | 4,556,713 |
| 28 | 12 and 19 and 27 | 1,869 |
| 29 | limit 28 to (human and english language) | 1,856 |

**Table S3** Systematic search strategies from APA PsycInfo

| **#** | **Query** | **Results from 09 Jul 2025** |
| --- | --- | --- |
| 1 | repeat*.ti,ab,id. | 118,375 |
| 2 | recurrent.ti,ab,id. | 23,863 |
| 3 | re?exposure.ti,ab,id. | 388 |
| 4 | subsequent.ti,ab,id. | 120,173 |
| 5 | post?index.ti,ab,id. | 46 |
| 6 | post?index.ti,ab,id. | 46 |
| 7 | Readmission.ti,ab,id. | 3,370 |
| 8 | re?admission*.ti,ab,id. | 4,249 |
| 9 | re?hospital*.ti,ab,id. | 2,063 |
| 10 | re?visit*.ti,ab,id. | 18,006 |
| 11 | revolv*.ti,ab,id. | 4,790 |
| 12 | 1 or 2 or 3 or 4 or 5 or 6 or 7 or 8 or 9 or 10 or 11 | 282,397 |
| 13 | "adverse drug reaction*".ti,ab,id. | 1,386 |
| 14 | "Drug related side effect*".ti,ab,id. | 125 |
| 15 | "adverse drug effect*".ti,ab,id. | 219 |
| 16 | "drug-related adverse reaction*".ti,ab,id. | 6 |
| 17 | "adverse drug event*".ti,ab,id. | 482 |
| 18 | 13 or 14 or 15 or 16 or 17 | 2,138 |
| 19 | Geriatric Patients/ | 14,701 |
| 20 | older adulthood/ | 25,362 |
| 21 | aged.ti,ab,id. | 317,838 |
| 22 | old*.ti,ab,id. | 622,971 |
| 23 | geriatric*.ti,ab,id. | 21,589 |
| 24 | senior*.ti,ab,id. | 34,256 |
| 25 | elder*.ti,ab,id. | 81,928 |
| 26 | "advanced age".ti,ab,id. | 1,971 |
| 27 | 19 or 20 or 21 or 22 or 23 or 24 or 25 or 26 | 844,165 |
| 28 | 12 and 18 and 27 | 67 |
| 29 | limit 28 to (human and english language) | 63 |

**Table S4** Systematic search strategies from IPA (Searched from inception to 08 July 2025)

| **Search ID** | **Query** | **Results** |
| --- | --- | --- |
| S1 | repeat* | 9078 |
| S2 | recurrent | 5281 |
| S3 | re?exposure | 0 |
| S4 | subsequent | 10318 |
| S5 | post?index | 1119 |
| S6 | re?admission* | 136 |
| S7 | re?hospital* | 799 |
| S8 | re?visit* | 65 |
| S9 | revolv* | 105 |
| S10 | S1 OR S2 OR S3 OR S4 OR S5 OR S6 OR S7 OR S8 OR S9 | 24252 |
| S11 | adverse drug reaction* | 23094 |
| S12 | Drug related side effect* | 172 |
| S13 | adverse drug effect* | 3971 |
| S14 | drug-related adverse reaction* | 92 |
| S15 | adverse drug event* | 3451 |
| S16 | S11 OR S12 OR S13 OR S14 OR S15 | 28674 |
| S17 | aged | 20359 |
| S18 | old* | 18985 |
| S19 | geriatric* | 13937 |
| S20 | senior* | 1272 |
| S21 | elder* | 10424 |
| S22 | advanced age | 619 |
| S23 | S17 OR S18 OR S19 OR S20 OR S21 OR S22 | 48514 |
| S24 | S10 AND S16 AND S23 | 175 |
| S25 | limit S24 to (human and english language) | 147 |

**Table S5** The methodologic quality of studies included in this study as assessed by JBI Critical Appraisal Checklist for Studies Reporting Prevalence or Incidence Data

| Joanna Briggs Institute (JBI) Critical Appraisal Checklist for Prevalence Studies | | | | | | | | | | |  | |  |  |
| --- | --- | --- | --- | --- | --- | --- | --- | --- | --- | --- | --- | --- | --- | --- |
| S.NO | Primary Author and year | 1 | 2 | 3 | 4 | 5 | 6 | 7 | 8 | 9 | | No. of 'Yes' responses | % of 'Yes' responses | ^¶^Study Quality |
|  |  | Was the sample frame appropriate to address the target population? | Were study participants sampled in an appropriate way? | ^¥^Was the sample size adequate? | Were the study subjects and the setting described in detail? | Was the data analysis conducted with sufficient coverage of the identified sample? | Were valid methods used for the identification of the condition? | Was the condition measured in a standard, reliable way for all participants? | Was there appropriate statistical analysis? | Was the response rate adequate, and if not, was the low response rate managed appropriately? | |  |  |  |
| 1 | Lopez et al., 2025 | Yes | Yes | Yes | Yes | Yes | Yes | Yes | Yes | Not applicable | | 8 | 77.8 | High |
| 2 | Hohl et al., 2019 | Yes | Yes | Unclear | No | Unclear | Yes | Yes | No | Not applicable | | 4 | 44.4 | Low |
| 3 | Lee et al., 2018 | Yes | Yes | Yes | Yes | Yes | No | Yes | Yes | Not applicable | | 7 | 77.8 | Low |
| 4 | Parameswaran Nair et al., 2017 | Yes | Yes | Yes | Yes | Yes | Yes | Yes | No | Not applicable | | 7 | 77.8 | High |
| 5 | Hauviller et al., 2016 | Yes | Yes | Yes | Yes | Yes | Yes | Yes | No | Not applicable | | 8 | 88.9 | High |
| 6 | Davies et al., 2010 | Unclear | Yes | Yes | No | Yes | Yes | Yes | No | Not applicable | | 5 | 55.5 | Moderate |
| 7 | Zhang et al., 2009 | Yes | Yes | Yes | Yes | Yes | Yes | Yes | No | Not applicable | | 7 | 77.8 | High |
| 8 | Zhang et al., 2007 | Yes | Yes | Yes | Yes | Yes | Yes | Yes | No | Not applicable | | 7 | 77.8 | High |
| 9 | Cooper, 1999 | No | Yes | No | Yes | No | Yes | Yes | No | Unclear | | 4 | 44.4 | Low |

^¶^The following cut-offs was used to assess the quality of individual studies: High quality (low risk): if 70% or more of the answers are "Yes", moderate quality (moderate risk): if 50% to 69% of the answers are "Yes", and low quality (high risk): if less than 50% of the answers are "Yes

^¥^A sample size of 100 and above was assumed as adequate sample size.

**Table S6** Risk of bias (ROB) assessment for the outcome at least one ADR-related hospitalisation using the ROB in non-randomised studies of exposure (ROBINS-E) tool

| First author (Year) | ROB domains | | | | | | |  |
| --- | --- | --- | --- | --- | --- | --- | --- | --- |
|  | Confounding | Selection of the participants | Classification of Exposure | Deviation from intended exposure | Missing data | Measurement of Outcomes | Selection of the Reported Result | Overall ROB |
| For the outcome repeat ADR-related hospitalisations | | | | | | | | |
| Lopez et al., 2025 | High | Low | Some concerns | Low | Some concerns | Some concerns | Some concerns | High |
| Parameswaran Nair et al., 2017 | Some concerns | Low | Low | Low | Some concerns | Low | Some concerns | Some concerns |
| Hauviller et al., 2016 | Some concerns | Some concerns | Some concerns | Low | Some concerns | Some concerns | Some concerns | Some concerns |
| Davies et al., 2010 | High | Some concerns | Some concerns | Low | High | Some concerns | Some concerns | High |
| Min Zhang et al. 2009 | Low | High | High | Low | Some concerns | Some concerns | Some concerns | High |
| Min Zhang et al. 2007 | High | High | High | Low | Some concerns | Some concerns | Some concerns | High |
| Cooper, 1999 | High | High | Some concerns | Low | Some concerns | High | Some concerns | High |
| **Overall** |  |  |  |  |  |  |  | **Serious** |
| For studies reporting repeat ADR frequency or number of patients with at least one repeat ADRs without records of ADR-related hospital admissions | | | | | | | | |
| Hohl et al., 2019 | High | Low | Low | Low | Some concerns | Low | Some concerns | High |
| Lee et al., 2018 | High | High | High | Low | Some concerns | Some concerns | Some concerns | High |
| **Overall** |  |  |  |  |  |  |  | **Serious** |

**Table S7** IR of repeat ADRs/associated hospitalisations

a. For studies reporting ADR-related hospitalisations

| First author (Year) | Number of patients with at least one ADR-related hospitalisations | No. of participants with index ADR-related hospitalisations | Follow-up durations in year | IR per 100 person-year^a^ |
| --- | --- | --- | --- | --- |
| Lopez et al., 2025 | 36 | 409 | 2 | 4.4 |
| Parameswaran Nair et al., 2017 | 15 | 112 | 1 | 13.4 |
| Hauviller et al., 2016 | 87 | 1000 | 1 | 8.7 |
| Davies et al., 2010 | 16 | 163 | 1 | 9.8 |
| Min Zhang et al. 2009 | 5056 | 28548 | 3 | 5.9 |
| Cooper, 1999 | 12 | 52 | 4 | 5.8 |

Abbreviations: ADR, Adverse Drug Reaction; IR, Incidence Rate. ^a^The figures are approximated to the nearest whole number in Fig 2.

Note: Some of the studies included for estimating incidence and IR of repeat ADR-related hospitalisation lacked detailed information on patients who died and/or were lost to follow-up between the index and repeat ADR-related hospitalisations (Davies et al., 2010, Lopez et al., 2025, Cooper, 1999); consequently, all the estimates assume complete follow-up and do not account for death as a competing risk.

b. For studies reporting repeat ADR frequency or number of patients with at least one repeat ADRs without records of ADR-related hospital admissions.

| First author (Year) | Repeat ADRs (Frequency or Patient Count) | Index ADRs (Frequency or Patient Count) | Follow-up duration (years) | IR per 100 person-year |
| --- | --- | --- | --- | --- |
| Lee et al., 2018 | 30520^a^ | 244173^b^ | 5 | 2.5 |
| Hohl et al., 2019 | 132^c^ | 330^d^ | NR | - |

^a^ Number of patients with at least one repeat ADRs

^b^ Number of patients with index ADRs

^c^ Frequency of repeat ADRs

^d^ Frequency of index ADRs

Abbreviations: ADR, Adverse Drug Reaction; IR, Incidence Rate

**Table S8** Repeat ADRs without hospitalisation records or those associated with rehospitalisation, as well as implicated drugs

| Study | Repeat ADRs | Implicated drugs as reported in the included studies | Implicated drugs classified based on ATC level 2 |
| --- | --- | --- | --- |
| Repeat ADRs associated with rehospitalisation | | | |
| Lopez et al., 2025 | Fall (27.8%, n=10), hemorrhagic events (16.7%, n=6), constipation (11.1%, n=4), confusion (8.3%, n=3)  anaemia (5.6%, n=2), hypoglycemia (5.6%, n=2)  convulsion (2.8%, n=1), chest pain (2.8%, n=1)  dyspnea (2.8%, n=1), encephalopathy (2.8%, n=1), incorrect administration routes (alimentary wrong way) (2.8%, n=1), suicidal ideation (2.8%, n=1), infection (2.8%, n=1), dizziness (2.8%, n=1), rhabdomyolysis (2.8%, n=1) | NR |  |
| Parameswaran Nair et al., 2017 | Acute kidney injury (44.4%, n=8), hypotension (11.1%, n=2), hyperkalaemia (11.1%, n=2), hypothyroidism (5.6%, n=1), diarrhoea (5.6%, n=1), hypoglycaemia (5.6%, n=1), syncope (5.6%, n=1), severe nausea (5.6%, n=1), increased lactate levels (5.6%, n=1) | Furosemide (27.6%, n=8), candesartan (13.7%, n=4), spironolactone (13.7%, n=4), ramipril (10.3%, n=3), metformin (6.9%, n=2), ethacrynic acid (3.4%, n=1), digoxin (3.4%, n=1), losartan (3.4%, n=1), amiodarone (3.4%, n=1), insulin (3.4%, n=1), irbesartan (3.4%, n=1), propranolol (3.4%, n=1), trimethoprim (3.4%, n=1) | Diuretics (44.8%, n=13), agents acting on the RAAS (31.0%, n=9), drugs used in cardiac therapy (6.9%, n=2), drugs used in diabetes (10.3%, n=3), beta blocking agents (3.4%, n=1), antibacterials (3.4%, n=1) |
| Hauviller et al., 2016 | Aplasia or agranulocytosis (60.2%, n=156, occurred in 57 patients, associated with 41 patient readmission), polyneuropathy (12%, n=24, occurred in 12 patients, associated with 5 patient readmission), bleeding events (6.2%, n=16), increase of INR (3.5%, n=9), increase of digoxinemia (1.5%, n=4) | Chemotherapy (76.4%, n=198), fluindione (n=10), standard heparin (n=4), enoxaparine (n=3), digoxin (n=3), dexamethasone (n=2), levodopa (n=2), warfarin (n=2), hydrocortisone (n=2), amiodarone (n=2), loxapine (n=2), prednisone (n=2), benzerazide (n=2), lysine (n=1), acetylsalicylate (n=1), duloxetine (n=1), cotrimoxazole (n=1), trihexyphénidyle (n=1), oxazepam (n=1), divalproate sodium (n=1), norfloxacine (n=1), ribavirine (n=1), tramadol (n=1), bromazepam (n=1), insuline glargine (n=1), citalopram (n=1), furosemide (n=1), ceftazidime (n=1), fluvastatine (n=1), rosuvastatine (n=1), rifampicine (n=1), allopurinol (n=1), linezolide (n=1) | Antineoplastic/immunosuppressants (76.4%, n=198), antithrombotic agents (7.9%, n=20), corticosteroid (2.4%, n=6), psycholeptics/psychoanaleptics (2.4%, n=6), drugs used in cardiac therapy (1.97%, n=5), antibacterials (1.97%, n=5), anti-parkinson drugs (1.2%, n=3), various (0.79%, n=2), lipid modifying agents (0.79%, n=2), blood substitutes and perfusion solutions (n=1), antiepileptics (n=1), antivirals (n=1), analgesics (n=1), drugs used in diabetes (n=1), diuretics (0.4%, n=1), antigout preparations (n=1) |
| Zhang et al., 2007  Australia | Nausea and/or vomiting (8.0%, n=816), haemorrhagic disorder (5.5%, n=557), drug-induce osteoporosis (4.8%, n=495), poisoning by cardiovascular agents (3.9%, n=397), dermatitis due to drugs (3.5%, n=355) | Antineoplastic/immunosuppressive drugs (11.0%), corticoids/synthetic analogues (10.1%), anticoagulants (8.6%), antirheumatics/nsaids (5.1%), and  opioids/related analgesics (4.9%). | Antineoplastic/immunosuppressive drugs (11.0%), corticosteroid (10.1%), antithrombotic agents (7.9%, n=20), analgesics (10.0%) |
| Cooper, 1999  USA | NSAIDs gastropathy (n=3), NSAID-associated bleeding (n=2), Oversedation due to multiple CNS depressants (n=1) | NR |  |
| Repeat ADRs without records of hospitalisation | | | |
| Hohl et al., 2019 | Constipation (9.8%, n=13), dizziness (7.5%, n=10), diarrhea (7.5%, n=10), rash (6.0%, n=9), hyponatremia (5.3%, n=8) | Hydrochlorothiazide (6.4%, n=13), furosemide (3.9%, n=8), oxycodone (3.4%, n=7), prednisone (3.4%, n=7), ramipril (3.0%, n=6), glyburide (3.0%, n=6), warfarin (3.0%, n=6), acetylsalicylic acid (3.0%, n=6) | Diuretics (10.3%, n=21), antithrombotic agents (6.0%, n=12), analgesics (3.4%, n=7), corticosteroids (3.4%, n=7), agents acting on RAAS (3.0%, n=6), drugs used in diabetes (3.0%, n=6) |

Abbreviations: ADR, Adverse Drug Reaction; IR, Incidence Rate; RAAS, Renin–Angiotensin–Aldosterone System

**Additional supplementary information**

**Figure S1** Leave-one-out meta-analysis result

**Median age grouped data when age is presented in interval** (<https://www.statology.org/median-of-grouped-data/>).

Median of Grouped Data = L + W [(N/2 – C) / F]

where:

L: Lower limit of median class

W: Width of median class

N: Total Frequency

C: Cumulative frequency up to median class

F: Frequency of median class
